# New Medical Journals

**Published:** 1845-07

**Authors:** 


### THE MEDICAL EXAMINER.

### PHILADELPHIA, JULY, 1845.

### NEW MEDICAL JOURNALS.

If the amount of medical intelligence disseminated in the form of books and journals be any indication of the reading habits of the physicians of a country,—and we think the inference is a fair one,—the profession in the United States is entitled to the greatest praise. In no other country in the world, in proportion to the population, are so many and excellent books published on the various branches of the science. In this respect, too, we are eminently eclectic. As remarked of us by a transatlantic cotemporary: "No sooner is a work of merit, or one from an author of reputation ushered into the world, than forthwith it is disseminated throughout the length and breadth of the New World." In the number of medical journals, we are in a fair way to exceed, not only any but all other nations put together. Already we can boast at least a dozen; and instead of every *lustrum*, almost every *lunation* brings us a new one. That all are adequately supported, we will not pretend to say. Some, doubtless, owe their existence less to the demand of readers than to the zeal of their projectors, who "persecute time with hope," and, perchance, "find no other advantage in the process, but only the losing of hope by time;" but, there are others again, whose well stored pages and lengthened existence leave no room to doubt that they are welcome visitors to extensive circles of subscribers. Very recently, three new aspirants have appeared, one in Canada, and two in the United States. The date of their several beginnings is in good keeping with the "go-ahead" spirit manifested in all American enterprises. They commence not with the first, the middle, or the end of the year; but, regardless of times and seasons, they "construe the times to their necessities," and start off, each at its own appointed time—one in April, one in May, and the other in June of this present year. The first of these in the order of time as well as of magnitude, is "THE BRITISH AMERICAN JOURNAL OF MEDICAL AND PHYSICAL SCIENCE," edited by Archibald Hall, M. D., printed and published by John C. Becket, Montreal. Each number contains "forty-eight demi-octavo pages of closely printed matter," and is issued monthly, at three dollars per annum. The objects of the undertaking are to aid in diffusing a knowledge of the facts and principles of Medical Science in the Provinces, and especially to afford a vehicle for the "valuable stores of information collected by the assiduous perseverance of scientific men" in that interesting region. Numbers *one*, *two* and *three* (for April, May and June) which we have received, are well stored with valuable matter, original and selected, and afford promise of the Journal being a spirited and well conducted publication.

